# Supplementary material for: Empiric azithromycin alters the upper respiratory microbiome and resistome without anti-inflammatory benefit in COVID-19
Source: Nat Microbiol. 2026 Mar 16;11(4):1100–12. doi: 10.1038/s41564-026-02285-8 (PMC13056551; doi:10.1038/s41564-026-02285-8)
Supplement: Supplementary file 2 — Reporting Summary [file 41564_2026_2285_MOESM2_ESM.pdf]

nature portfolio

Corresponding author(s): Charles R. Langelier

Last updated by author(s): 01/14/2026

Reporting Summary

Nature Portfolio wishes to improve the reproducibility of the work that we publish. This form provides structure for consistency and transparency in reporting. For further information on Nature Portfolio policies, see our Editorial Policies and the Editorial Policy Checklist.

Statistics

For all statistical analyses, confirm that the following items are present in the figure legend, table legend, main text, or Methods section.

n/a	Confirmed	
☐	☒	The exact sample size (*n*) for each experimental group/condition, given as a discrete number and unit of measurement
☐	☒	A statement on whether measurements were taken from distinct samples or whether the same sample was measured repeatedly
☐	☒	The statistical test(s) used AND whether they are one- or two-sided<br>*Only common tests should be described solely by name; describe more complex techniques in the Methods section.*
☐	☒	A description of all covariates tested
☐	☒	A description of any assumptions or corrections, such as tests of normality and adjustment for multiple comparisons
☐	☒	A full description of the statistical parameters including central tendency (e.g. means) or other basic estimates (e.g. regression coefficient) AND variation (e.g. standard deviation) or associated estimates of uncertainty (e.g. confidence intervals)
☐	☒	For null hypothesis testing, the test statistic (e.g. *F*, *t*, *r*) with confidence intervals, effect sizes, degrees of freedom and *P* value noted<br>*Give P values as exact values whenever suitable.*
☒	☐	For Bayesian analysis, information on the choice of priors and Markov chain Monte Carlo settings
☒	☐	For hierarchical and complex designs, identification of the appropriate level for tests and full reporting of outcomes
☐	☒	Estimates of effect sizes (e.g. Cohen's *d*, Pearson's *r*), indicating how they were calculated

Our web collection on statistics for biologists contains articles on many of the points above.

Software and code

Policy information about availability of computer code

Data collection	Metatranscriptomic data was processed using the open-source CZ ID pipeline (https://czid.org/). Microbiome profiling was performed within CZ ID using the Illumina mNGS pipeline (v7.1). Resistome profiles were generated using the CZ ID AMR pipeline (v0.2), which leverages the Resistance Gene Identifier (RGI) tool and the Comprehensive Antibiotic Resistance Database (CARD).
Data analysis	All code was written in R v4.2.3 or R 4.0.3. Data processing was performed using R packages dplyr (v1.1.4) and tidyverse (v2.0.0). Plots were generated using R packages ggplot2 (v3.5.1), ggpubr (v0.6.0), scales (1.3.0) and ggbeeswarm (v0.7.2). Package versions for each analysis are reported in the code repository.

For manuscripts utilizing custom algorithms or software that are central to the research but not yet described in published literature, software must be made available to editors and reviewers. We strongly encourage code deposition in a community repository (e.g. GitHub). See the Nature Portfolio guidelines for submitting code & software for further information.

Data

Policy information about availability of data

All manuscripts must include a data availability statement. This statement should provide the following information, where applicable:

- Accession codes, unique identifiers, or web links for publicly available datasets
- A description of any restrictions on data availability
- For clinical datasets or third party data, please ensure that the statement adheres to our policy

Data used in this study is available at ImmPort Shared Data under the accession number SDY1760 and in the NLM's Database of Genotypes and Phenotypes (dbGaP) under the accession number phs002686.v2.p2. Source data for each figure are provided with this manuscript in the Source Data file. All code is deposited in the following Bitbucket repository: https://bitbucket.org/kleinstein/impacc-public-code/src/master/azithromycin_manuscript/ .

Research involving human participants, their data, or biological material

Policy information about studies with human participants or human data. See also policy information about sex, gender (identity/presentation), and sexual orientation and race, ethnicity and racism.

Reporting on sex and gender	Sex at birth is reported for each study participant.
Reporting on race, ethnicity, or other socially relevant groupings	Self-identified race is reported for each patient, and includes the following groups: Asian, Black, White, Multiple, Other, American Indian/Alaska Native and Native Hawaiian/Pacific Islander.<br><br>Self-identified hispanic ethnicity is reported for each patient.
Population characteristics	A comprehensive table of population characteristics is provided in Supplemental Table 1. Briefly, the median age in the cohort was 59.0 (20.0). 61% were male. All participants were hospitalized for COVID-19. 474 participants received no antibiotics, 366 participants received azithromycin and 324 participants received other antibiotics.
Recruitment	IMPACC is a prospective longitudinal study that enrolled 1164 patients hospitalized for COVID-1923–25,38,39 from 20 hospitals across 15 academic biomedical centers within the U.S. between May 2020 and March 2021. Inclusion criteria included: 1) age ≥ 18 years; 2) confirmed SARS-CoV-2 positivity by reverse transcription PCR (RT-PCR) testing; and 3) confirmed understanding by participant and/or surrogate of the data to be collected and the study procedures, and willingness to participate in the surveillance cohort. Exclusion criteria included: 1) underlying medical problems which, in the opinion of the investigator, may have been associated with mortality unrelated to COVID-19 within 48 hours of hospitalization; 2) a decision by the patient or surrogate prior to hospitalization to limit care to comfort measures; or 3) medical problems or conditions such as pregnancy which might impact interpretation of the immunologic data obtained. The Department of Health and Human Services Office for Human Research Protections (OHRP) and NIAID concurred that the IMPACC study qualified for public health surveillance exemption. The study protocol was reviewed by each site's institutional review board (IRB), with twelve sites conducting as a public health surveillance study, and three sites integrating the IMPACC study into IRB-approved protocols (The University of Texas at Austin, IRB 2020-04-0117; University of California San Francisco, IRB 20-30497; Case Western Reserve University, IRB STUDY20200573) with participants providing informed consent. Participants enrolled at sites operating as a public health surveillance study were provided information sheets describing the study including the samples to be collected and plans for analysis and data de-identification. Participants who requested not to participate after review of the study plan and information were not enrolled. Participants were not compensated while hospitalized but were subsequently compensated for outpatient visits and surveys. This study was registered at clinicaltrials.gov (NCT04378777) and followed the Strengthening the Reporting of Observational Studies in Epidemiology (STROBE) guidelines.
Ethics oversight	The Department of Health and Human Services Office for Human Research Protections (OHRP) and NIAID concurred that the IMPACC study qualified for public health surveillance exemption. The study protocol was reviewed by each site's institutional review board (IRB), with twelve sites conducting as a public health surveillance study, and three sites integrating the IMPACC study into IRB-approved protocols (The University of Texas at Austin, IRB 2020-04-0117; University of California San Francisco, IRB 20-30497; Case Western Reserve University, IRB STUDY20200573) with participants providing informed consent. Participants enrolled at sites operating as a public health surveillance study were provided information sheets describing the study including the samples to be collected and plans for analysis and data de-identification. Participants who requested not to participate after review of the study plan and information were not enrolled.

Note that full information on the approval of the study protocol must also be provided in the manuscript.

Field-specific reporting

Please select the one below that is the best fit for your research. If you are not sure, read the appropriate sections before making your selection.

☒ Life sciences ☐ Behavioural & social sciences ☐ Ecological, evolutionary & environmental sciences

For a reference copy of the document with all sections, see nature.com/documents/nr-reporting-summary-flat.pdf

Life sciences study design

All studies must disclose on these points even when the disclosure is negative.

Sample size	No sample size calculation was preformed as we leveraged all available data from the large (N=1164) IMPACC observational cohort. Many microbiome and resistome studies have a much smaller population size, and our sample size was robust enough to identify several significant findings.
Data exclusions	Data from every patient was analyzed. Negative control water samples were processed in parallel with the participant samples, and we used previously described negative binomial model to exclude microbes and antimicrobial resistance genes likely to be contaminants from the laboratory environment. Based on the results of this model, microbial taxa and antimicrobial resistance genes that were present at a significantly higher abundance in participant samples than in negative controls (FDR < 0.1) were retained for downstream analyses.
Replication	No external metatranscriptomic dataset with azithromycin exposure data exists and is available for replication, to our knowledge.
Randomization	This was an observational study and thus no randomization was carried out.
Blinding	This was an observational study and thus blinding to intervention group was carried out.

Reporting for specific materials, systems and methods

We require information from authors about some types of materials, experimental systems and methods used in many studies. Here, indicate whether each material, system or method listed is relevant to your study. If you are not sure if a list item applies to your research, read the appropriate section before selecting a response.

Materials & experimental systems

n/a	Involved in the study	
☒	☐	Antibodies
☒	☐	Eukaryotic cell lines
☒	☐	Palaeontology and archaeology
☒	☐	Animals and other organisms
☒	☐	Clinical data
☒	☐	Dual use research of concern
☒	☐	Plants

Methods

n/a	Involved in the study	
☒	☐	ChIP-seq
☒	☐	Flow cytometry
☒	☐	MRI-based neuroimaging

Plants

Seed stocks	*Report on the source of all seed stocks or other plant material used. If applicable, state the seed stock centre and catalogue number. If plant specimens were collected from the field, describe the collection location, date and sampling procedures.*
Novel plant genotypes	*Describe the methods by which all novel plant genotypes were produced. This includes those generated by transgenic approaches, gene editing, chemical/radiation-based mutagenesis and hybridization. For transgenic lines, describe the transformation method, the number of independent lines analyzed and the generation upon which experiments were performed. For gene-edited lines, describe the editor used, the endogenous sequence targeted for editing, the targeting guide RNA sequence (if applicable) and how the editor was applied.*
Authentication	*Describe any authentication procedures for each seed stock used or novel genotype generated. Describe any experiments used to assess the effect of a mutation and, where applicable, how potential secondary effects (e.g. second site T-DNA insertions, mosiacism, off-target gene editing) were examined.*
